# Prostate cancer incidence in men with prostate‐specific antigen below 3 ng/mL: The Finnish Randomized Study of Screening for Prostate Cancer

**DOI:** 10.1002/ijc.34274

**Published:** 2022-09-15

**Authors:** Idris Olasunmbo Ola, Kirsi Talala, Teuvo Tammela, Kimmo Taari, Teemu Murtola, Paula Kujala, Jani Raitanen, Anssi Auvinen

**Affiliations:** ^1^ Institute of Medicine, Sahlgrenska Academy Gothenburg University Gothenburg Sweden; ^2^ Finnish Cancer Registry Helsinki Finland; ^3^ Department of Urology TAYS Cancer Center Tampere Finland; ^4^ Faculty of Medicine and Health Technology Tampere University Tampere Finland; ^5^ Department of Urology Helsinki University Hospital Helsinki Finland; ^6^ Medical Faculty University of Helsinki Helsinki Finland; ^7^ Department of Pathology Fimlab Laboratories Tampere Finland; ^8^ Faculty of Social Sciences Tampere University Tampere Finland; ^9^ UKK Institute for Health Promotion Research Tampere Finland

**Keywords:** cancer screening, prostate cancer incidence, prostate‐specific antigen, randomized controlled trials, rescreening intervals

## Abstract

Prostate‐specific antigen (PSA)‐based screening for prostate cancer (PCa) can reduce PCa mortality, but also involves overdetection of low‐risk disease with potential adverse effects. We evaluated PCa incidence among men with PSA below 3 ng/mL and no PCa diagnosis at the first screening round of the Finnish Randomized Study of Screening for PCa. Follow‐up started at the first screening attendance and ended at PCa diagnosis, emigration, death or the common closing date (December 2016), whichever came first. Cox regression analysis was used to estimate hazard ratios and their confidence intervals (CI). Among men with PSA <3 ng/mL, cumulative PCa incidence was 9.1% after 17.6 years median follow‐up. Cumulative incidence was 3.6% among men with baseline PSA 0 to 0.99 ng/mL, 11.5% in those with PSA 1.0 to 1.99 ng/mL and 25.7% among men with PSA 2 to 2.99 ng/mL (hazard ratio 9.0, 95% CI: 7.9‐10.2 for the latter). The differences by PSA level were most striking for low‐risk disease based on Gleason score and EAU risk group. PSA values <1 ng/mL indicate a very low 20‐year risk, while at PSA 2 to 2.99 ng/mL risks are materially higher, with 4‐ to 5‐fold risk for aggressive disease. Using risk‐stratification and appropriate rescreening intervals will reduce screening intensity and overdetection. Using cumulative incidence of clinically significant PCa (csPCa) as the criterion, rescreening intervals could range from approximately 3 years for men with initial PSA 2 to 2.99 ng/mL, 6 years for men with PSA 1 to 1.99 ng/mL to 10 years for men with PSA <1 ng/mL.

## Graphical abstract

What's new?

Prostate‐specific antigen (PSA)‐based screening can reduce prostate cancer mortality, but it may also involve overdetection of low‐risk disease with potential adverse effects. Here, the authors evaluated prostate cancer risk among men with initial PSA levels below 3 ng/mL during 20 years of follow‐up in the Finnish Randomized Study of Screening for Prostate Cancer. Prostate cancer incidence increased with the initial PSA level, especially for low‐risk disease, which represented the majority of cases. The authors suggest tailoring prostate cancer screening intervals by the initial PSA level to reduce overdetection and unnecessary treatment of indolent prostate cancers.

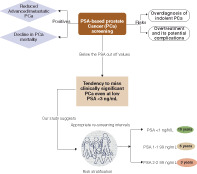

**Abbreviations**
5‐ARI: 5‐alpha reductase inhibitor
aHR: adjusted hazard ratio
CI: confidence interval
csPCa: clinically significant prostate cancer
EAU: European Association of Urology
ERSPC: European Randomized Study of Screening for Prostate Cancer
FinRSPC: Finnish Randomized Study of Screening for Prostate Cancer
HR: hazard ratio
mpMRI: multiparametric magnetic resonance imaging
PCa: prostate cancer
PLCO: prostate, lung, colorectal and ovarian cancer screening
PSA: prostate‐specific antigen
SII: Social Insurance Institution
USPSTF: United States Preventive Services Task Force

## 1 INTRODUCTION

Prostate cancer (PCa) is the second most common cancer in men and accounts for 7.1% of all cancer cases globally.^1^

The prostate‐specific antigen (PSA) was introduced as a biomarker, but soon also adopted for PCa screening. Since the advent of PSA‐based screening for PCa around 1990, there has been a major increase in PCa diagnosis in most industrialized countries, but also a decline in PCa mortality partly due to improvement in treatment and likely also widespread PSA screening.^2^ Empirical evidence has also demonstrated harm from PSA‐based screening, which includes false‐positive results that may require additional biopsy, and more importantly, overdiagnosis and overtreatment of low‐risk disease involving potential complications.^3^

The overall effectiveness of prostate cancer screening with PSA has been widely debated, especially the balance between the benefit of PCa mortality reduction relative to the risks of overdiagnosis and overtreatment, as well as overall cost‐effectiveness and impact on quality of life.^4^, ^5^

The Prostate, Lung, Colorectal and Ovarian Cancer Screening (PLCO) trial in the United States has not shown a clear PCa mortality reduction from annual PCa screening in follow‐up up to 17 years.^6^, ^7^ The major setbacks were the high rate of contamination in the control arm of the study and the low biopsy compliance among the screen‐positive participants.^6^, ^8^ In contrast, after 16 years of follow‐up, the larger European Randomized Study of Prostate Cancer (ERSPC) has consistently showed a 20% relative reduction in PCa mortality, with absolute mortality risk reduction up to 0.18% (one death per 570 invited men).^9^, ^10^ A large cluster‐randomized trial in the United Kingdom involved only a single screen and showed no mortality impact at 10 years.^11^

The United States Preventive Services Task Force (USPSTF) gave routine PCa screening a “Grade D” assessment in 2012, recommending against screening for all ages and categories of men.^12^ This was followed by a decline in incidence of low‐risk PCa, but also a steady rise in metastatic PCa in the United States in 2012 to 2015.^13^ The revised USPSTF recommendation in 2018 took a more lenient position, assigning a “C” grade and delegated the decision to screen to men aged 55 to 69 years and their physicians, with a “Grade D” assigned for men 70 years and older to indicate that the risks outweigh the benefits.^3^

Initial PSA values during screening have been shown to predict PCa development, including tumor grade and stage.^14^ In a Swedish study, the 20‐year cumulative incidence of PCa was as high as 40.3% among men with PSA 2 to 2.99 ng/mL.^15^ A similar trend was observed in the Rotterdam^16^ and Aarau^17^ components of the ERSPC.

PSA values of 4 ng/mL and above are generally taken as indication for further diagnostic tests in screening. However, values lower than 3 ng/mL have remained contentious, demanding careful considerations to avoid the risk of overdiagnosis and unnecessary treatment of clinically insignificant PCa, while also avoiding missing clinically relevant PCa.

The aim of our study was to evaluate the incidence of both clinically significant and low‐risk PCa among men with initial PSA levels below 3 ng/mL in the Finnish Randomized Study of Prostate Cancer (FinRSPC) during 20 years of follow‐up. We also aimed to suggest appropriate rescreening intervals based on risk levels for clinically relevant PCa by baseline PSA.

## 2 MATERIALS AND METHODS

The FinRSPC is a population‐based randomized trial that began in 1996 as a component of the larger ERSPC study.^18^ A total of 80 458 men aged 55 to 67 years and living in the Helsinki and Tampere metropolitan areas were identified from the Finnish Population Registry. Randomization took place in 1996 to 1999, with 8000 men annually allocated into the intervention arm and the remaining approximately 12 000 men in the control (usual care) arm, giving a ratio of approximately 1:1.5. The men in the screening group were invited for serum PSA determination every 4 years until age 71 (excluding men diagnosed with prostate cancer and those who emigrated from the study region).

Our study was a retrospective cohort study within the FinRSPC trial. We evaluated men who were randomized into the screening arm and had initial PSA levels <3 ng/mL at the first screening attendance in the FinRSPC trial.

The study population was stratified into three categories by the initial PSA concentration (PSA <1 ng/mL, PSA 1 to 1.99 ng/mL and PSA 2 to 2.99 ng/mL). Men with higher PSA were excluded. Up until 1998, the FinRSPC protocol used digital rectal examination and from 1999 onward, free vs total PSA ratio with a cut‐off of 16% as an auxiliary test for men with PSA between 3.0 and 3.99 ng/mL. Men with a PSA of 4.0 ng/mL or above were referred to urological clinics for diagnostic tests including a digital rectal examination, transrectal ultrasonography and prostate biopsy.^18^

The follow‐up started at the first screening attendance and ended on the date of emigration, PCa diagnosis, death or the common closing date in the end of 2016, whichever came first.

Information on family history of prostate cancer was extracted using a questionnaire at the initial screening.

The Finnish Cancer Registry provided information on PCa diagnoses. PSA level at PCa diagnosis, clinical staging and Gleason score at diagnosis were obtained from medical records. The EAU risk classification based on PSA, Gleason score and clinical stage was used to stratify prostate cancer into low, intermediate and high risk, as well as advanced disease groups.^19^

Data on comorbidity was based on the Deyo modification of the Charlson Comorbidity Index^20^ after excluding prostate cancer. The index was derived from the diagnoses obtained from the Finnish National Care Register for Health Care for 1996 to 2000.^20^ Due to its established lowering effect on PCa risk, a separate analysis was conducted of men with diabetes.^21^ The criterion for diabetes was at least one purchase of antidiabetic medication between 1996 and 1999 recorded in the Social Insurance Institution's (SII) prescription database.

Data on finasteride/dutasteride (5‐alpha reductase inhibitors [5‐ARI]) use at baseline were also extracted from the national prescription database of the SII.^22^ Five‐ARI, a medication used for benign prostatic hyperplasia was included in the analysis as a covariate, because it lowers PSA levels and can result in false negative results masking the effect of a PCa.

Inferential statistics were obtained using the Cox proportional hazards model with hazard ratios (HRs) as measures of association between PSA level and PCa incidence. The proportionality assumption was checked using the Schoenfeld and scaled Schoenfeld residuals, which showed significant overall global test (*P*‐value .026). However, the individual components of the model were not statistically significant. Therefore, we retained the model with the predictors. Interactions between PCa incidence and predictor variables were evaluated by comparing goodness of fit between nested models with and without interaction terms using a *P*‐value <.05 based on a likelihood ratio test as a criterion. All data were analyzed using Stata version 16.1.

Family history of PCa in a first‐degree relative, comorbidity, diabetes and use of 5‐ARI were used as covariates. However, in the final model, we excluded family history, comorbidity and diabetes, because they did not improve the model fit.

Prostate cancer diagnosis was explored as subgroups of the primary outcome by Gleason score and EAU risk classification. PCa with a Gleason score of <7 (or low risk group by EAU classification) is considered clinically insignificant PCa since it predicts a nonaggressive clinical course. Gleason 7 and higher (or intermediate, high and advanced risk groups by EAU classification), on the other hand, indicates a medium to high‐grade clinically significant disease (CsPCa) with a higher risk of progression.

To evaluate the impact of PSA mediated by screening, a secondary analysis was performed utilizing screen‐detected PCa as the outcome variable. Also, participation at the subsequent (second and third) screening rounds by PSA level was analyzed.

The yield for csPCa in the second round of the FinRSPC, which was roughly 0.5%,^23^ was used as a reference value in the determination of suitable rescreening intervals.

## 3 RESULTS

A total of 20 268 men were included in the analysis after excluding those with PSA 3 ng/mL or higher, those invited for screening but failed to show up, and those randomized but not invited for screening (Table 1). The mean age at enrollment was 59.8 years (SD 4.4). Of the study population, 7.1% (N = 1429) had a family history of prostate cancer in a first‐degree relative and 8.8% (1756) had any comorbidity (Charlson comorbidity index >0; Table 1).

**TABLE 1:** Description of the FinRSPC study population of men with PSA <3 ng/mL at initial screen

Variables	Category	Frequency N (%)	PSA <1 ng/mL	PSA 1‐1.99 ng/mL	PSA 2‐2.99 ng/mL	Prostate cancer		
						All PCa N (%)	csPCA (Gleason ≥7)	csPCa (EAU > low risk group)
Family history	Yes	1429 (7.1)	677	543	209	202 (14.1)	92 (6.4)	109 (7.6)
	No	18 774 (92.6)	10 006	6437	2331	1634 (8.7)	878 (4.7)	999 (5.3)
	Missing	65 (0.3)						
Comorbidity score	0	18 197 (89.8)	9603	6304	2290	1714 (9.4)	912 (5.0)	1035 (5.7)
	1+	1756 (8.7)	948	592	216	126 (7.2)	61 (3.5)	75 (4.3)
	Missing	315 (1.5)						
5‐ARI use	Yes	248 (1.2)	108	76	64	39 (15.7)	27 (10.9)	29 (11.7)
	No	20 020 (98.8)	10 611	6924	2485	1801 (9.0)	946 (4.7)	1081 (5.4)
Total		20 268 (63.7)	10 719	7000	2549	1840 (9.1)	973 (4.8)	1110 (5.5)

Of the study participants, 52.9% (10719) had a baseline PSA level < 1 ng/mL, 34.5% (7000) 1 to 1.99 ng/mL and 12.6% (2549) 2 to 2.99 ng/mL (Table 1).

The participants were followed up for on average 20.7 years, with a median follow‐up of 17.6 years. Of the subgroups, the median follow‐up time was roughly 17 years for the men with PSA <2 ng/mL (17.8 for those with <1 ng/mL and 17.3 for men with PSA 1‐1.99 ng/mL) and somewhat shorter for men with higher PSA (15.4 years for men with initial PSA 2‐2.99 ng/mL).

Approximately 20% of men with initial PSA <3 ng/mL failed to attend further screening after the first round. Attendance at subsequent rounds was somewhat higher in men with initial PSA <1 ng/mL than in the other subgroups, with 52% (5571) of those men completing all the three rounds compared to 47% (3253) for men with PSA 1 to 1.99 ng/mL and nearly 34% (858) for those with PSA 2 to 2.99 ng/mL.

A total of 1840 PCa cases were diagnosed with a mean age of 71 years at diagnosis (SD 6.2). Of the PCa cases, low EAU risk cases constituted 38%, intermediate‐risk cases 32%, high‐risk disease 12% and advanced disease 18%. The crude PCa incidence rate was approximately 6 per 1000 person‐years, corresponding to a cumulative incidence of 9.1%.

The cumulative PCa incidence among men with the initial PSA <1 ng/mL was 3.6%, compared to 11.5% among those with PSA 1 to 1.99 ng/mL and 25.7% in men with 2 to 2.99 ng/mL (Table 2).

**TABLE 2:** Prostate cancer (PCa) incidence and hazard ratios for PCa by baseline PSA

	Number of PCa cases and incidence rate (per 1000 person‐years)^a^	Serum PSA at initial screen (ng/mL)		
		0‐0.99 aHR (95% CI)	1‐1.99 aHR (95% CI)	2‐2.99 aHR (95% CI)
Number of cases (%)^b^		382 (3.6)	803 (11.5)	655 (25.7)
*All PCa*	1840 (6.0)	1 (reference)	3.4 (3.1‐3.9)	9.0 (7.9‐10.3)
*Clinically insignificant PCa*				
Gleason<7	867 (2.8)	1 (reference)	4.9 (4.0‐6.0)	17.1 (13.9‐21.0)
EAU low risk	678 (2.2)	1 (reference)	5.6 (4.5‐7.3)	19.5 (15.3‐24.9)
*Clinically significant PCa subgroups*				
Gleason 7	597 (1.9)	1 (reference)	3.3 (2.7‐4.1)	6.6 (5.3‐8.2)
Gleason>7	376 (1.2)	1 (reference)	2.1 (1.7‐2.7)	3.9 (3.0‐5.1)
EAU intermediate risk	577 (1.9)	1 (reference)	3.8 (3.1‐4.7)	9.2 (7.3‐11.6)
EAU high risk	209 (0.7)	1 (reference)	2.3 (1.7‐3.3)	4.8 (3.3‐6.9)
EAU advanced disease	324 (1.0)	1 (reference)	2.2 (1.7‐2.8)	3.8 (2.9‐5.1)
*5‐ARI use*				
5‐ARI users	39 (0.1)	1 (reference)	1.0 (0.4‐2.3)	2.4 (1.2‐5.0)
5‐ARI non‐users	1801 (5.8)	1 (reference)	3.5 (3.1‐4.0)	9.3 (8.2‐10.5)

^a^ PCa incidence showed the crude total number of PCa cases recorded for each variable over the follow‐up period. The incidence rate (per 1000 person‐years) was calculated using the total number of PCa for each variable per total accrued time at risk for all study participants.

^b^ Percentage PCa cases per total participants in each PSA category.

Compared to men with initial PSA <1 ng/mL, the age‐ and 5‐ARI ‐adjusted risk of PCa in men with PSA 1 to 1.99 ng/mL was roughly 3‐fold (adjusted hazard ratio aHR 3.4, 95% CI: 3.0‐3.8) and 9‐fold in men with PSA 2 to 2.99 ng/mL (aHR 8.8, 95% CI: 7.7‐10.0; Table 2).

When PSA was explored as a continuous predictor of PCa, for each unit (ng/mL) increase in PSA, there was on average a 3‐fold increase in risk of PCa irrespective of the clinical significance (aHR: 3.2, 95% CI: 3.0‐3.4). Addition of a squared term of PSA in the model showed a strong curvilinear relationship (aHR 10.3, 95% CI: 7.5‐14.0), demonstrating stronger risk per unit rise in PSA level toward the higher range of the PSA values.

In the analysis of PCa by Gleason score, the gradient by baseline PSA was most striking for clinically insignificant cases with Gleason score <7 (aHR: 17.1, 95% CI: 13.9‐21.0 for PSA 2‐2.99 ng/mL, compared to PSA <1 ng/mL) and with the smallest magnitude of effect for Gleason >7 cancer (aHR: 3.9, 95% CI: 3.0‐5.1 for PSA 2‐2.99 ng/mL compared to PSA <1 ng/mL; Table 2).

In the analysis where PCa aggressiveness was classified by the EAU risk group, the findings were comparable to those by Gleason grade. A very steep gradient by PSA was found for low‐risk disease, with substantially lower aHRs for high‐risk and advanced PCa (Table 2).

The cumulative incidence of clinically significant PCa (Gleason 7 and above) over time varied strongly by baseline PSA. Cumulative incidence reached 0.5% for men with PSA 2 to 2.99 before 4 years, among men with PSA 1 to 1.99 at 6 years, while in men with PSA <1, at 10 years (Table 3). A similar pattern was observed for csPCa defined by EAU risk groups other than the low‐risk group (Table 4).

**TABLE 3:** Clinically significant prostate cancer (Gleason score 7 or higher) (csPCa) cases and cumulative incidence by duration of follow‐up and baseline PSA level

Follow‐up time (years)	Cumulative number of csPCa cases and cumulative csPCa incidence by Gleason score		
	<1 ng/mL	1‐1.99 ng/mL	2‐2.99 ng/mL
2	1 (0.01%)	3 (0.04%)	1 (0.04%)
4	3 (0.03%)	16 (0.23%)	22 (0.9%)
6	9 (0.08%)	34 (0.5%)	70 (2.8%)
8	19 (0.18%)	77 (1.1%)	102 (4.0%)
10	49 (0.5%)	161 (2.3%)	147 (5.8%)

**TABLE 4:** Clinically significant prostate cancer (EAU risk group other than low risk) (csPCa) cases and cumulative incidence by duration of follow‐up and baseline PSA level

Follow‐up time (years)	Cumulative number of csPCa cases and cumulative csPCa incidence by EAU risk		
	<1 ng/mL	1‐1.99 ng//mL	2‐2.99 ng/mL
2	1 (0.01%)	4 (0.06%)	3 (0.12%)
4	3 (0.03%)	21 (0.3%)	29 (1.14%)
6	8 (0.07%)	44 (0.6%)	93 (3.7%)
8	18 (0.17%)	95 (1.4%)	139 (5.5%)
10	49 (0.5%)	188 (2.7%)	193 (7.6%)

There was an interaction between PSA level and use of 5‐ARI (*P* < .001). Among 5‐ARI users, the risk doubled with PSA >2 compared to men with PSA <1 ng/mL, but no increase in men with PSA 1 to 1.99 ng/mL (aHR: 0.97, 95% CI: 0.4‐2.3). A similar but more pronounced effect was observed among the nonusers, where the risk increased sharply for all PSA categories with the largest effect in men with PSA 2 to 2.99 ng/mL (aHR: 9.0, 95% CI: 7.9‐10.3).

In a secondary analysis limited to screen‐detected PCa as the endpoint, the gradient with initial PSA was extremely steep (aHR: 10.2, 95% CI: 7.3‐14.4 for PSA 1‐1.99 ng/mL and aHR: 41.0, 95% CI: 29.3‐57.5 for PSA 2‐2.99 ng/mL).

## 4 DISCUSSION

The results indicate that cumulative incidence of PCa among men with initial PSA levels <3 ng/mL is substantial: up to 9.1% after 20 years of follow‐up in the FinRSPC study. The risk of PCa increases sharply with the initial PSA level and demonstrates a supra‐linear relationship when explored as a continuous predictor.

The total number of participants decreased with each subsequent screening round for a variety of reasons, including reaching the stopping age 71 years when men were no longer invited for screening in the FinRSPC. This resulted in fewer subsequent screening rounds associated with higher PSA levels at the initial screen.

Of the PCa cases, three quarters were Gleason 7 or lower and two thirds were EAU low and intermediate risk. This suggests that only a moderate proportion represents clinically significant disease.

Since most screening protocols have used cut‐off values 3 to 4 ng/mL as indication for diagnostic assessment, this threshold means missing some clinically significant cancers, which needs to be evaluated against the additional costs and potential for overdiagnosis with a lower threshold, or an ancillary test for men with intermediate PSA values.

The finding of increased risk with PSA level is in tandem with the findings of previous studies.^16^, ^17^, ^24^ In our study, the distribution of PCa incidence of 3.6%, 11.5% and 25.7%, respectively, among men with PSA <1, 1 to 1.99 and 2 to 2.99 ng/mL shows the increasing risk with each unit (ng/mL) rise in PSA levels. These figures are similar in pattern, though only about half in magnitude compared to those reported by Frånlund et al,^15^ showing 20‐year cumulative incidence of 7.9%, 26.0% and 40.3%, respectively, at PSA levels <1, 1 to 1.99 and 2 to 2.99 ng/mL in the Gothenburg component of the ERSPC trial.

Moreover, the findings show that low PSA levels predict mostly low‐risk, clinically insignificant PCa, as the hazard ratios decreased toward higher Gleason and EAU risk categories. This was also evident in the larger differentials in incidence rates for low‐risk disease with PSA level than for higher grade PCa. Overall, our findings reiterate the concern that the current protocol of PSA‐based screening could increase overdiagnosis and overtreatment of indolent disease.

For most tests, optimal thresholds are usually defined, but gray zones could also exist, below which risks can be safely assumed low and above which examinations are justified. For this intermediate group (gray zone), procedures may need to be adapted by measures such as ancillary testing and adjusted re‐screening intervals. Several recommendations suggest longer screening intervals for men with low initial PSA levels.^25^

The rationale for adjusting the screening protocol by PCa risk (risk‐adapted or personalized screening) is intuitively appealing. However, there is no straight forward method for estimating the optimal screening interval. The yield (risk of screen‐detected cancer or incidence proportion) in the FinRSPC, as well as the ERSPC as a whole has been 2% to 4% per screening round for any type of PCa.^18^ For clinically significant PCa (Gleason 7 and higher) the detection rate in the first and second round of the FinRSPC was approximately 0.5%.^23^ A simple approach to select an appropriate re‐screening interval would be to define the interval as the time duration required to accrue the average level of risk of clinically significant PCa for all screening participants.

However, since sensitivity of PSA is well below 100%, that is, not all cases would be detected by screening, a higher risk level would need to be used to estimate the accumulated level of risk corresponding to that among all screened men. A broad range of PSA sensitivities have been reported, with estimates ranging from about 24% when compared to biopsy,^26^ to over 87% when screen‐negative participants are compared to the control arm in the ERSPC.^27^, ^28^

Therefore, for a hypothetical PSA sensitivity of 80%, for example, it will take up to 15 years, 7 years and 3 to 4 years, respectively, for PSA <1, 1 to 1.99 and 2 to 2.99 ng/mL to reach the hypothetical detection rate of 3.75% (derived as a ratio of the overall yield [3%] divided by PSA sensitivity taken as 0.8) for any type of PCa. The estimated screening interval to detect any PCa would increase to 18 years, 8 years and 4 years, respectively, for the PSA groups if PSA sensitivity was 60%.

According to our study, the optimal screening window to detect clinically significant PCa based on the cumulative risk for clinically significant PCa (Gleason score ≥7) in the FinRSPC would be approximately 3 years for initial PSA values 2 to 2.99 ng/mL, 6 years for PSA 1 to 1.99 ng/mL and 10 years for PSA <1 ng/mL.

Adjusting a screening protocol for men with low initial PSA values could also minimize over‐detection, while still reducing advanced PCa.^17^ A re‐screening interval of 6 to 8 years has been suggested for men with PSA <1 ng/mL, 3 to 4 years at PSA 1 to 1.99 ng/mL and 1 to 2 year for men with PSA 2 to 2.99 ng/mL.^17^

A modeling study suggested that PSA‐stratified screening can substantially reduce the testing burden and modestly reduce overdiagnosis, while preserving most of the mortality reduction.^29^ This approach is being used in the ongoing ProScreen trial in Finland^30^ and the European Association of Urology and the American Cancer Society also incorporate such approach in their guidelines.

To minimize the risk of over‐detection or false‐positive results in a PSA‐based screening system, various adjunct methods have also been introduced to better detect clinically significant PCa and reduce unnecessary invasive confirmatory tests following PSA screening. PSA‐based techniques include PSA velocity, PSA doubling time, PSA density of the transition zone of the prostate and percentage free PSA.

The use of additional biochemical parameters such as other kallikreins in Prostate Health Index and *4Kscore*© have proved useful in minimizing invasive confirmatory procedures. Urinary markers including *Progensa©*, PCA3, the PSA precursor Pro‐PSA, Mi‐Prostate score© and *ExoDx©* prostate have also shown promise of enhancing PSA‐based screening.^31^, ^32^ Furthermore, multiparametric magnetic resonance imaging (mp‐MRI) has been widely adopted in the diagnostic pathway to reduce unnecessary detection of clinically insignificant disease.^5^

An important concern, albeit not often considered, is the significant cost and healthcare resource requirements these adjunct tests might add to a screening program if used on a large scale.^15^

### 4.1 Study limitations and strengths

Our study used high‐quality prospective data collected within the FinRSPC trial, which is the largest component in the ERSPC trial. We achieved complete follow‐up due to register‐based design and were able to compile comprehensive clinical data from medical records on practically all cases. Due to the large number of men in the screening arm of the FinRSPC, the statistical power of the study was also high.

In the evaluation of men with 5‐ARI use, the power of the study was limited due to the small number of 5‐ARI users. A further limitation is that we did not have information on prostate volumes, which would have allowed calculation of PSA density values.

## 5 CONCLUSION

Our study among men with low initial PSA levels in the FinRSPC trial after 17.6 median follow up years shows that the risk of prostate cancer progressively increases with PSA even at levels below 3 ng/mL.

Tailoring screening intervals by initial PSA level may reduce over‐detection, unnecessary treatments and unnecessary costs of indolent prostate cancers. These could range from approximately 3 years for men with baseline PSA 2 to 2.99 ng/mL, 6 years at PSA level 1 to 1.99 ng/mL and 10 years for men with PSA <1 ng/mL based on duration of follow‐up needed to accrue a similar cumulative incidence of csPCa as detectable at the next screening round.

## AUTHOR CONTRIBUTIONS

**Anssi Auvinen:** Proposed the study topic and conceptualized the analysis as well as designed the methodology; obtained funding; supervision. **Idris Olasunmbo Ola:** Conducted the data‐analyses as well as prepared the first article draft and revised it based on co‐author comments. **Kirsi Talala:** Data curation. **Jani Raitanen:** contributed to data analysis. **Teuvo Tammela:** contributed to study conduct and data generation; obtained funding. **Kimmo Taari:** contributed to study conduct and data generation. **Teemu Murtola:** contributed to data curation and generation. **Paula Kujala:** contributed to study conduct and data generation. All authors contributed to critical review and final approval. The work reported in the article has been performed by the authors, unless clearly specified in the text.

## CONFLICT OF INTEREST

Idris Ola, Kirsi Talala, Jani Raitanen, Teuvo Tammela and Paula Kujala have no conflicts of interest to disclose. Anssi Auvinen has received a lecture fee from Amgen and Janssen, Kimmo Taari received research funding from Medivation/Astellas/Pfizer, Orion, Myovant. Teemu Murtola received consultant fees from Novartis, Janssen, Astellas, Amgen; lecture fees from Ferring, Novartis, Janssen, Sanofi, Bayer, Roche, Pfizer, Astellas and Ipsen; and research funding from Bayer and Orion.

## ETHICS STATEMENT

The FinRSPC trial study design was reviewed by the Institutional Review Boards of Helsinki University Hospital (March 12, 1996) and Tampere University Hospital (tracking number # R10167). A written informed consent was obtained from study participants in the screening arm of the trial.

National Institute for Health and Welfare (Dnro THL/1182/14.06.00/2021) has provided a research permission.

## Data Availability

The FinRSPC is a component of the ERSPC, and the primary data can be released as part of the European trial at their website (www.erspc.org). The FinRSPC data used for our study can be requested from the investigators for analysis.
